# Family and home correlates of children's physical activity in a multi-ethnic population: the cross-sectional child heart and health study in england (CHASE)

**DOI:** 10.1186/1479-5868-8-11

**Published:** 2011-02-15

**Authors:** Alison M McMinn, Esther MF van Sluijs, Claire M Nightingale, Simon J Griffin, Derek G Cook, Chris G Owen, Alicja R Rudnicka, Peter H Whincup

**Affiliations:** 1 Medical Research Council Epidemiology Unit, Cambridge, UK; 2 Community Health Sciences, St George's University of London, London, UK

## Abstract

**Background:**

The influence of the family and home environment on childhood physical activity (PA) and whether this differs between ethnic groups remains uncertain. This paper investigates associations between family and home factors and childhood PA in a multi-ethnic population and explores whether associations differ between ethnic groups.

**Methods:**

Cross-sectional study of 9-10 year-old schoolchildren, in which PA was objectively measured by Actigraph GT1 M accelerometers for ≤7 days to estimate average activity counts per minute (CPM). Information on 11 family and home environmental factors were collected from questionnaires. Associations between these factors and CPM were quantified using multi-level linear regression. Interactions with ethnicity were explored using likelihood ratio tests.

**Results:**

2071 children (mean ± SD age: 9.95 ± 0.38 years; 47.8% male) participated, including 25% white European, 28% black African-Caribbean, 24% South Asian, and 24% other ethnic origin. Family PA support and having a pet were associated with higher average CPM (adjusted mean difference: 6 (95%CI:1,10) and 13 (95%CI:3,23), respectively) while car ownership and having internet access at home were associated with lower average CPM (adjusted mean difference: -19 (95%CI:-30,-8) and -10 (95%CI:-19,0), respectively). These associations did not differ by ethnicity. Although the number of siblings showed no overall association with PA, there was some evidence of interaction with ethnicity (p for ethnicity interaction = 0.04, 0.05 in a fully-adjusted model); a positive significant association with number of siblings was observed in white Europeans (per sibling CPM difference 10.3 (95% CI 1.7, 18.9)) and a positive non-significant association was observed in black African-Caribbeans (per sibling CPM difference: 3.5 (-4.2, 11.2)) while a negative, non-significant association was observed in South Asians (per sibling CPM difference -6.0 (-15.5, 3.4)).

**Conclusions:**

Some family and home environmental factors have modest associations with childhood PA and these are mostly similar across different ethnic groups. This suggests that targeting these factors in an intervention to promote PA would be relevant for children in different ethnic groups.

## Background

Higher levels of physical activity in childhood are associated with a reduced risk of developing obesity [1] and having other metabolic [2] and cardiovascular disease [3] risk factors. Additionally, it has been associated with beneficial effects on mental health [4,5]. Promoting physical activity in children is therefore recognised as an important public health issue. It is currently recommended that children participate in at least 60 minutes of moderate-to-vigorous physical activity per day to gain benefits to health but there is evidence that an appreciable proportion of children (defined as under the age of 12 years) are not meeting this target, particularly girls[6,7]. There has also recently been evidence from UK studies that South Asian children are less active than other ethnic groups [8,9]. These findings suggest that girls and South Asian children may be priority groups for interventions to promote physical activity.

To develop effective interventions to promote physical activity, it is important to have a good understanding of the influences on children's physical activity to identify which factors should be targeted in an intervention. It is also important to explore whether these influences are consistent across different population groups to establish whether a generic intervention is appropriate or whether interventions should be tailored to specific populations. Some studies have investigated whether the correlates of physical activity differ between boys and girls and indicate some level of consistency in associations between genders [10-12] but less attention has been paid to possible ethnic differences in influences on physical activity. The few studies that have been conducted on this topic have tended to focus on adolescents, are mostly based in North America or Australia and have studied different ethnic or racial groups making comparisons difficult [13-18]. Additionally, much of this previous research has used self-reported measures of physical activity, known to have poorer validity than objective measures.

The aim of the current study was to investigate the correlates of 9-10-year-old children's objectively measured physical activity in a multi-ethnic UK population and assess whether any associations between these factors and children's physical activity differ between ethnic groups. We focused specifically on factors related to family influences and home circumstances as these are potentially important influences on children's physical activity [19] and we hypothesised that the prevalence of these factors and associations with physical activity might vary across ethnic groups.

## Methods

### Study design & participants

The analyses presented in this paper are based on the Child Heart And health Study in England (CHASE). This is a population-based, cross-sectional study, set up to investigate the health of children of white European, South Asian (including Indian, Pakistani, Bangladeshi, and other South Asian) and black African-Caribbean origin. The study was conducted in the cities of London, Birmingham and Leicester. Ethical approval for this study was obtained from the relevant Multicentre Research Ethics Committee and it complies with the Helsinki Declaration. Details on the sampling strategy have been described elsewhere [9]. In brief, two separate random samples, each of 100 schools, was selected, the first from schools that had a high prevalence (20-80%) of South Asian pupils, the second from schools with a high prevalence of black African-Caribbean pupils. Schools that declined to participate (30%) were replaced with schools from the same sampling frame with a similar ethnic composition and in the same or a neighbouring borough. From the 200 schools, one or two classes (depending on school size) of Year 5 (9-10 year-old) school children were invited to participate. The measurements of physical activity and its potential determinants were carried out in 78 schools in the final phase of the study (January 2006-February 2007). From these schools, 3449 Year 5 children were invited to participate and 2393 (69% response) provided written parental consent and participated.

### Measures

A team of trained research assistants visited each participating school to make physical measurements, administer questionnaires and initiate physical activity monitor assessment. A parent questionnaire was also distributed to children to take home. Physical activity was measured using the Actigraph GT1 M accelerometer (ActiGraph, LLC, Pensacola, FL, USA), a one-dimensional movement sensor, validated in children using indirect calorimetry [20] and doubly-labelled water methods [21]. Children were shown how to wear the monitor on a belt around the waist and were asked to wear it during waking hours for seven consecutive days, except when participating in water-based activities. The accelerometer was set to record activity counts at 5-second intervals. The data were processed using a dedicated software programme (MAHUFFE - available from http://www.mrc-epid.cam.ac.uk) to remove periods of twenty minutes or more that had continuous zero activity counts. Participants with an average daily recording time of less than 600 minutes and less than one day of recording were excluded from analyses. The outcome variable used here is average activity counts per minute. Participants with implausible values (< 50 or >2000 average counts per minute) were excluded.

Ethnicity was parent-reported and defined using standard approaches [22]. Based on this, children were categorised as white European, black African-Caribbean, South Asian, and other. Height and weight were collected using standardised protocols; height was measured to the last complete millimetre using a CMS stadiometer and weight to the nearest 0.1 kg using Tanita TBF-300A scales. Both instruments were calibrated regularly throughout the study. From these, ponderal index was calculated (kg/m^3^) and used for descriptive purposes. Ponderal index was used as a marker of body fatness instead of BMI because of its greater independence of height, an issue of particular importance when making ethnic comparisons of body fatness[23].

Information on 10 factors related to family and home circumstances was gathered from the child questionnaire and one further variable, parental social class, was derived from questions in the parent questionnaire (see Table 1). Previously used questions were used to measure social class [24] and the social support variables (family PA support and family encouragement) [25,26]. The family social support variables were divided in this way due to a previous confirmatory factor analysis which showed that these variables had evidence of good fit when grouped into these two factors[27]. All the child questions for this analysis were pilot tested in a sample of 20 children from two schools (10 in each) participating in the CHASE study prior to the main data collection and minor amendments were made following this. However, no major difficulties in answering these questions were identified.

**Table 1:** Description of family and home environment variables

Variable	Description and coding
Socioeconomic status (SES)	Based on mother's or father's occupation (depending on who is highest) using SOC 2000 coding [24]. Divided into three categories: low/medium/high.
Car ownership	Family car ownership. Coding: yes/no.
Number of parents	Number of parents reported as living in the household. Coding: two/less than two (the latter category included the 1% of participants who reported living with neither parent).
Number of siblings	Number of siblings reported living in the household. Coding: 0/1/2/≥3.
Household density	Total number of people reported living in the household divided by the number of bedrooms reported in the house.
Family PA encouragement	Sum of responses (yes/no) to 2 questions about whether family members encourage the child to do physical activity and tell the child that physical activity is good for their health. Based on distribution of responses, dichotomised into high (answered yes to both questions) or low.
Family PA support	Sum of responses (yes/no) to 3 questions about whether family members take the child somewhere to do physical activity, watch the child participate in physical activity and do physical activity with the child. Score range 0-3.^1^
Pets	Whether or not child has a pet at home. Coding: yes/no.
Garden	Whether or not child has a garden at home (any outdoor space attached to the home, either for private use or shared with neighbours). Coding: yes/no.
Internet access	Whether or not child has access to the internet at home. Coding: yes/no.
Cable TV	Whether or not child has cable television at home. Coding: yes/no.

1. Participants with only one answer missing had this answer imputed with 'no'; this was done for 1.5% of participants. Participants with 2 or more answers missing were coded as missing for this variable.

Due to the high prevalence of having a television at home (97.3% of children answered yes), this variable was excluded from analyses.

### Statistical analyses

All statistical analyses were conducted using Stata Version 10 (StataCorp LP, College Station, Texas, 2007). Physical activity was measured in counts per minute. To take account of measurement error in levels of physical activity, regression calibration was used to derive an average level of physical activity for each child providing at least one day of measurement. This method takes into account the within-child variation in physical activity across a variable number of days of recording, allows for variation in activity by day of week, and clustering within school to provide an unbiased average of counts per minute for each child [28,29].

Descriptive statistics (means or percentages) were provided for each of the variables of interest for each ethnic group and formal statistical tests for differences between ethnic groups were carried out. Corrected chi-squared tests were used for categorical variables and linear regression models were used for continuous variables; in both cases these were adjusted for clustering of children within school.

Associations between each family/home environment factor and physical activity (counts per minute) were examined using multilevel linear regression models to allow for clustering of children within schools. All models were adjusted for sex, age quartiles, month of measurement (strongly related to physical activity levels in this study population [9]), ethnicity and a random effect for school. Variables which were statistically significant were then reassessed in a mutually adjusted model which included all statistically significant family/home environment factors in the same multilevel model. To examine whether the associations between home/family circumstances and physical activity differed by ethnic group, we also examined the statistical significance of interactions between each of the study variables and ethnicity in models adjusting for sex, age quartiles, month of measurement and clustering of children within school using likelihood ratio tests. Any significant interaction terms were added into the mutually adjusted model with all the significant family/home environment variables.

## Results

### Descriptive results

Among the 2393 children who participated, 2144 wore an accelerometer and 2071 had valid physical activity data, representing a 60% response rate from the total invited to participate. Only a small proportion of participants had only 1-2 days of physical activity data (11%) and this proportion did not differ markedly between ethnic groups. Participants excluded from analyses were more likely to be male than female (57.7% versus 47.8%, p = 0.001) but no significant differences were found in relation to age, ethnicity, ponderal index or parent social class. The ethnic composition of the sample was 24.5% white European, 27.8% black African-Caribbean, 23.5% South Asian, and 24.1% other ethnicity. As has previously been reported in this study[9], South Asian participants were significantly less active than the other ethnic groups (see Table 2). There were also significant differences in the distribution of most of the family and home circumstances variables between ethnic groups (see Table 2). For example, the proportion of participants living with two parents differed significantly across the ethnic groups (p < 0.001) with South Asian participants reporting the highest proportion (91%) and black African-Caribbean participants reporting the lowest (63%). Socio-economic position also differed significantly across the groups (p < 0.001) with a greater proportion of South Asian participants being in the lowest socioeconomic group (57%) compared to black African-Caribbeans (44%) and white Europeans (39%). White European participants were most likely to have pets (61% versus 20% of black African-Caribbean participants and 17% of South Asian participants, p < 0.001 for difference across groups) while black African-Caribbean participants were most likely to report having cable television at home (76% versus 72% of South Asian participants and 69% of white European participants, p < 0.05 for difference across groups).

**Table 2:** Means and percentages for physical measurements, physical activity and family and home circumstances variables^1^

Variable^2^	All (n = 2071)	White European (n = 508)	Black African-Caribbean (n = 576)	South Asian (n = 487)	Other (n = 500)	p^1^
Age (years)	9.95 ± 0.38	9.96 ± 0.38	9.89 ± 0.40	10.01 ± 0.36	9.93 ± 0.39	*
Gender (% boys)	47.8	49.6	47.4	47.8	46.4	
Ponderal index^3 ^(kg/m^3^)	13.16	13.29	13.27	12.83	13.21	†
PA (CPM)	481 ± 106	493 ± 105	492 ± 102	452 ± 108	484 ± 107	‡
SES						
- High	27.3	32.2	31.9	19.5	24.8	‡
- Medium	26.0	29.1	23.8	23.9	27.3	
- Low	46.7	38.7	44.3	56.6	47.9	
% own car	75.3	75.9	71.1	79.3	75.6	
% live with 2 parents	76.7	77.1	62.6	91.1	78.0	‡
N siblings (0-3+)	1.7 ± 1.0	1.4 ± 0.9	1.8 ± 1.0	2.0 ± 0.9	1.5 ± 1.0	‡
Household density	1.7 ± 0.7	1.6 ± 0.6	1.8 ± 0.7	1.9 ± 0.8	1.7 ± 0.6	‡
Family PA encouragement						
- Low	37.3	30.0	34.8	44.1	40.9	‡
- High	62.7	70.0	65.2	55.9	59.1	
Family PA support (0-3)	2.1 ± 1.0	2.2 ± 0.9	2.1 ± 1.0	2.0 ± 1.0	2.2 ± 0.9	‡
% with pets	33.9	60.6	19.6	16.8	40.0	‡
% with garden	73.0	80.0	63.7	75.1	74.4	†
% with internet access	65.7	70.9	65.3	60.2	66.1	*
% with cable TV	71.3	68.9	76.0	72.1	67.5	*

1. All means, proportions and p-values for ethnic differences are adjusted for clustering within school. P-value code: *p < 0.05 †p < 0.01 ‡p < 0.001

2. CPM: counts per minute; SES: socio-economic status; PA: physical activity; TV: television.

3. Geometric means shown for log transformed variable.

### Associations between family and home factors and children's physical activity

When examined individually, five family and home factors were statistically significantly associated with children's physical activity (see Table 3). Owning a car and having internet access at home were associated with lower levels of physical activity, while living with less than two parents, the presence of family PA support and having pets at home were associated with higher levels of physical activity. In the mutually adjusted model, three of the five factors remained significantly associated with children's physical activity - family PA support, car ownership and having pets at home - while having internet access at home just failed to retain statistical significance (see Table 3).

**Table 3:** Associations between factors in the family and home environment and children's physical activity (counts/minute)

Variable	Level	All available data^1^				Mutually adjusted model^1^			
		N	Difference in CPM (95% CI)		p^2^	N	Difference in CPM (95% CI)		p^2^
SES	High	558	*ref*						
	Medium	530	-4	(-15, 7)	0.42				
	Low	953	2	(-8, 12)	0.67				
N parents	2	1423	*ref*			1222	*ref*		
	<2	432	12	(2, 22)	0.02	375	8	(-3, 19)	0.18
N siblings	Score 0-3+	1855	1	(-4, 5)	0.74				
Household density	Continuous	1835	-5	(-11, 1)	0.12				
Family PA encouragement	Low	659	*ref*						
	High	1108	8	(-1, 16)	0.10				
Family PA support	Score 0-3	1805	6	(1, 10)	0.01	1597	6	(1, 10)	0.01
Car ownership	No	507	*ref*			383	*ref*		
	Yes	1544	-22	(-31, -13)	<0.0001	1214	-19	(-30, -8)	<0.001
Garden at home	No	557	*ref*						
	Yes	1504	0	(-9, 10)	0.94				
Cable TV at home	No	588	*ref*						
	Yes	1458	3	(-6, 12)	0.51				
Internet access at home	No	708	*ref*			511	*ref*		
	Yes	1354	-12	(-20, -3)	0.006	1086	-10	(-19, 0)	0.06
Pets at home	No	1326	*ref*			1039	*ref*		
	Yes	679	13	(4, 22)	0.01	558	13	(3, 23)	0.01

1. All models adjusted for gender, age quartiles, month, ethnicity and a random effect for school. In the mutually adjusted model, differences are additionally adjusted for all other factors for which results are displayed in the final column.

2. P-values are comparisons with the reference (ref) group (except for continuous variables).

### Effect modification by ethnicity

No significant interactions with ethnicity were identified for the four factors included in the mutually adjusted model. However, although number of siblings showed no overall association with physical activity, a formal test for interaction between number of siblings and ethnicity was marginally statistically significant (p = 0.04, p = 0.05 in the mutually adjusted model with all significant factors). This appeared to reflect the difference between the significant positive association between number of siblings and CPM in white Europeans (per sibling CPM difference 10.3 [95% CI 1.7, 18.9]), the non-significant positive association in black African-Caribbeans (per sibling CPM difference: 3.5 [-4.2, 11.2]) and the non-significant inverse association in South Asians (per sibling CPM difference -6.0 [-15.5, 3.4]). No other statistically significant interactions with ethnicity were identified.

## Discussion

This study aimed to assess the family and home correlates of children's physical activity in a multi-ethnic UK population and investigate whether these associations differ by ethnic group. Family PA support, having pets at home, and owning a car were found to be significantly and independently associated with children's total physical activity and these associations were consistent between ethnic groups. The only difference in association between ethnic groups showed that, for white European participants, having siblings was associated with doing more physical activity, whereas a weak, non-significant negative association was observed in South Asian participants. These findings suggest that some family factors and home circumstances may have a role to play in shaping children's physical activity and that these seem to apply similarly to children of different ethnic groups.

The significant positive association between family PA support and children's physical activity found here is consistent with the Youth Physical Activity Promotion Model [19] which identifies family support as an important influence on children's physical activity. In general, previous findings on this association have been somewhat mixed [30-32], but a study in 9-10 year-old Danish children also reported a significant positive association between this type of support and objectively measured physical activity [10]. It suggests that this type of support (characterised by more practical support than verbal encouragement) may be helpful to increase children's physical activity. However, the modest size of the association observed suggests that this should only be targeted within a broader intervention to promote physical activity.

Family car ownership was found to be negatively associated, whereas family pet ownership was positively associated, with physical activity. The association with family car ownership is in slight contrast to the findings of Broderson et al[33] who reported that adolescent girls in a UK multi-ethnic population were more active in higher socio-economic groups (with car ownership being a component of this) although no association was found in boys. However, car ownership has been shown to be negatively associated with active travel in Australian children [34,35] and it could be through this behaviour that it impacts on total physical activity levels [36], which might explain the finding reported here. While this needs to be confirmed in further research, the finding in this study potentially supports the need for interventions to promote active travel in children. The positive association with pet ownership confirms an earlier observation from the CHASE study that dog ownership is associated with higher levels of physical activity in children [37]. The analyses presented here show that an association with pet ownership persists following adjustment for other family-related factors, suggesting that the association is independent of the other factors included in this analysis. However, due to the cross-sectional nature of this study, it is impossible to comment on the direction of the association (it could be that active children are more likely to own pets). Longitudinal research is therefore needed to further investigate this issue in more detail.

Having access to the internet at home was negatively associated with physical activity although this just fell short of significance (p = 0.06) once adjusted for the other variables that were significantly associated with physical activity. This may imply that children with internet access spend more time on the computer than those who do not, which in turn negatively impacts on their physical activity. It may not specifically be having internet access at home but simply computer usage that is associated with lower levels of physical activity. This is supported by some previous studies that have shown computer usage to be associated with low levels of physical activity [38] or sedentary behaviour [39] in children. This may support the theory that the time children spend on the computer displaces physical activity; however there is some dispute over this theory [40]. Nevertheless, encouraging children to spend less time on the computer in favour of physical activity may be an effective strategy.

The only factor showing a difference in association across ethnic groups was the reported number of siblings, although the interaction term was only of borderline significance. The significant positive association observed in white European children is supported by previous work in Australia, which included mostly white participants [41,42]. This association may well reflect the greater motivation and opportunities to be physically active associated with the presence of children. Further research is needed to confirm whether a negative association in South Asian children exists as the finding in this study was non-significant and, if so, explore potential explanations.

Although few differences in the family and home correlates of physical activity across ethnic groups were found in this study, it is possible that differences in the prevalence of these correlates (and other correlates not investigated here) between ethnic groups could contribute to the different physical activity levels observed. Indeed, as reported in Table 2, South Asian children reported slightly lower levels of social support, were less likely to have a pet at home and were more likely to report having a car compared to the other ethnic groups. While the findings suggest that we may want to target the same factors in efforts to increase children's physical activity across ethnic groups, it is possible that different strategies might be needed to address some of these factors. For example, a different approach for increasing family PA support may be needed for South Asian families compared to white European families. This is something that needs to be explored in future research, both quantitative and qualitative.

### Strengths and limitations

Giving strength to the findings presented here is the fact that an objective measure of physical activity was used which is known to have greater validity than self- or parent-report measures [43]. This is in contrast to much previous research on correlates of physical activity [30], particularly those studies that have investigated differences in correlates between ethnic groups [13-18]. We took an inclusive approach in dealing with the physical activity data to allow as many children as possible to be included in the analysis, maximizing response rates and limiting the potential bias resulting from participant exclusion. Our approach took into account the within-child variation (i.e. measurement error) in physical activity across a variable number of days of recording and day of the week to provide an unbiased average of physical activity for each child. It inherently gives more weight to children with more days of data in estimating both the fixed effects for day order and day of the week and includes random effects for both school and child. Our method therefore maintains sample representativeness whilst providing a robust, unbiased estimate of the average level of physical activity for each child. A sensitivity analysis that excluded children with only 1-2 days of physical activity data showed that the findings were not materially changed by this exclusion.

This study was carried out in 9-10 year-old children, an age-group in which a considerable proportion of children have been found to not meet the physical activity guidelines[7], with levels of activity being particularly low in South Asian children [9], and in which low physical activity levels are associated with adverse cardiometabolic consequences[3,29]. It is therefore important to identify correlates of physical activity in this age group to aid the development of future interventions to promote physical activity in this age group.

The large sample size is also a strength of this study, although it did not allow for detailed separate examination of the South Asian subcategories. The questions used to measure the family support variables were based on previously validated questions to help ensure that these were capturing the constructs of interest, although we acknowledge that we did not validate these questions in this particular study population and it is possible that their validity may differ between ethnic groups. However, the pilot testing of these questions (in a mixed-ethnic group) did not identify any major difficulties for the children completing them. It is important to acknowledge that only a selection of family and home factors were included in this analysis and the strengths of the associations identified were relatively small, implying that there are a number of other influences on children's physical activity. It is therefore possible that differences between ethnic groups exist for other correlates of physical activity not included in this analysis. Lastly, the cross-sectional nature of the study is a limitation as it does not allow for the investigation of causation. However, as there have been very few studies on this topic and certainly none conducted in UK children, these findings add valuable knowledge and can help inform future efforts to increase physical activity.

## Conclusions

Some family and home factors were found to be significantly associated with children's physical activity in this study. These associations were mostly consistent between different ethnic groups. These findings suggest that public health efforts to increase physical activity in children might not need to be tailored to specific ethnic groups. However, further research investigating ethnic differences in other correlates of children's physical activity would be beneficial. Additionally, it is possible that different approaches may be needed to target the same factors in different ethnic groups, such as family support, and this requires further exploration. Finally, as the findings presented here are cross-sectional, confirmation of these results in longitudinal studies is needed to determine whether targeting these factors in an intervention to promote physical activity in a multi-ethnic population would be worthwhile.

## Competing interests

The authors declare that they have no competing interests.

## Authors' contributions

PHW, DGC and CGO were involved in the acquisition of funding for and the setting up and running of the CHASE Study. AMM, EMvS, SJG, PHW, CGO and DGC were involved in the acquisition of data for this analysis. AMM, EMvS, SJG developed the research question and analysis design; all other authors provided input on the analysis design. AMM and CMN conducted the analysis with support from ARR. AMM drafted the manuscript. All authors were involved in interpreting the data and revising the manuscript and all have approved the final version for publication.
